# Survival of the Hmong population diagnosed with colon and rectal cancers in the United States

**DOI:** 10.1002/cam4.7087

**Published:** 2024-03-11

**Authors:** Margaret R. Walker, Kha Lor, Kajua B. Lor, Roberto J. Vidri, John M. Hampton, Cinthya Maldonado, Andrea M. Schiefelbein, Noelle K. LoConte

**Affiliations:** ^1^ Department of Medicine University of Wisconsin School of Medicine and Public Health Madison Wisconsin USA; ^2^ University of Wisconsin School of Medicine and Public Health Madison Wisconsin USA; ^3^ Medical College of Wisconsin School of Pharmacy Milwaukee Wisconsin USA; ^4^ Division of Surgical Oncology, Department of Surgery University of Wisconsin School of Medicine and Public Health Madison Wisconsin USA; ^5^ University of Wisconsin Carbone Cancer Center Madison Wisconsin USA; ^6^ Morgridge Institute for Research Madison Wisconsin USA

**Keywords:** colorectal cancer, Hmong community, National Cancer Database

## Abstract

**Background:**

The Hmong population constitutes an independent ethnic group historically dispersed throughout Southeast Asia; fallout from the Vietnam War led to their forced migration to the United States as refugees. This study seeks to investigate characteristics of the Hmong population diagnosed with in colorectal cancer (CRC) as well as survival within this population.

**Methods:**

Cases of colon and rectal adenocarcinoma diagnosed between 2004 and 2017 were identified from the National Cancer Database (NCDB). Summary statistics of demographic, clinical, socioeconomic, and treatment variables were generated with emphasis on age and stage at the time of diagnosis. Cox‐proportional hazard models were constructed for survival analysis.

**Results:**

Of 881,243 total CRC cases within the NCDB, 120 were classified as Hmong. The average age of Hmong individuals at diagnosis was 58.9 years compared 68.7 years for Non‐Hispanic White (NHW) individuals (*p* < 0.01). The distribution of analytic stage differed between the Hmong population and the reference NHW population, with 61.8% of Hmong individuals compared to 45.8% of NHW individuals with known stage being diagnosed at stage III or IV CRC compared to 0, I, or II (*p* = 0.001). However, there was no difference in OS when adjusting for potential confounders (HR 1.00 [0.77–1.33]; *p* = 0.998).

**Conclusions:**

Hmong individuals are nearly a decade younger at the time of diagnosis of CRC compared to the NHW individuals. However, these data do not suggest an association between Hmong ethnicity and overall survival, when compared to the NHW population.

## 1 INTRODUCTION

The Hmong population constitutes an independent ethnic group historically dispersed throughout Southeast Asia. Hmong communities have traditionally been self‐sustaining, agrarian, and animistic.^1^ Numerous Hmong individuals operated as secret soldiers under the direction of the US Central Intelligence Agency during the Vietnam War; resulting local hostility forced many to flee to the United States from their homes as refugees in one of the first waves of immigration.^2^ The Hmong community continues to grow and maintain extensive heterogeneity (as with any population) within the United States through multiple waves of mass migration, all while preserving rich cultural, language, and health‐seeking traditions. Based on 2017 American Community Survey data (the most recent year included in this study), an estimated 296,069 Hmong individuals resided in the United States with California, Minnesota, and Wisconsin home to the largest communities^3^. Prior observational studies signal higher incidences of infection‐related cancers (gastric, hepatic, cervical, and nasopharyngeal) in Hmong individuals despite an overall lower age‐adjusted incidence of cancer. When cancer is detected in this community, it is typically diagnosed at a later stage.^4^, ^5^, ^6^ Observations of Minnesota and California state cancer registries suggest a lower incidence of colorectal cancer (CRC) in the Hmong population.^4^, ^5^

Within the United States as a whole, CRC is the third most common newly diagnosed and deadly cancer in both men and women.^7^ Even more concerning, multiple studies have identified a national trend towards increasing incidence of higher‐stage CRC diagnosed at younger ages.^8^, ^9^, ^10^, ^11^, ^12^, ^13^ This observation may be partially explained by both physiology—enrichment of left‐sided colon tumors, molecular/histological differences, increased incidence malignancy driven by heritable mutations—as well as a lack of population‐wide screening at younger ages.^14^ Current preventative strategies include the use of colonoscopy for direct visualization and removal of precancerous lesions as well as stool‐based tests for detection of blood and cancer‐related DNA.^15^ The US Preventative Service Task Force has a grade B recommendation for screening at age 45–49,^15^ and the American Cancer Society recommends starting CRC screening at age 45 for average risk individuals.^16^ Prior work with Southeast Asian American communities demonstrated lower CRC screening rates among this population, and Hmong individuals, compared to other Asian American sub‐populations, displayed lower levels of CRC knowledge and a lower intention to obtain routine screening.^17^, ^18^, ^19^ Little is known about the demographics and survival of the Hmong population diagnosed with CRC in the United States.

This study evolved from conversations with individual Hmong‐American healthcare providers, Hmong leaders and Hmong community organizations who noted an increase in early‐onset of colon and rectal cancers within their communities. The objectives of this study were to summarize demographic, socioeconomic, and clinical traits of the Hmong population diagnosed with CRC using a national sample as well as to investigate the association between the Hmong race/ethnicity and overall survival (OS) of CRC.

## 2 METHODS

### 2.1 Data source and study population

This is a retrospective cohort study comprised of individuals diagnosed with colon and rectal cancers between 2004 and 2017 as identified from the National Cancer Database (NCDB). The NCDB collects data from approximately 1500 hospitals accredited by the Commission on Cancer (CoC) and captures an approximated 70% of cancers diagnosed within the United States.^20^, ^21^ Cancer cases were limited to those with only one CoC site reporting data and adenocarcinoma pathology (histology codes: 8140, 8141, 8143, 8144, 8145, 8147, 8150, 8154, 8160, 8161, 8163, 8190, 8200, 8201, 8210, 8211, 8213, 8220, 8221, 8230, 8243, 8250, 8254, 8260, 8261, 8310, 8320, 8323, 8380, 8401, 8410, 8440, 8460, 8470, 8490, 8500, 8503, 8510, 8265, 8507).^22^

Race and ethnicity were separately recorded for each patient into one of 30 race categories and one of 10 ethnicity categories (Spanish/Hispanic origin). We wish to emphasize that race is a sociopolitical construct and not equated with biological differences. Further NCDB's Spanish/Hispanic origin designation is a separate construct, and those of Spanish or Hispanic origin can also be of any racial origin. We chose to condense race and ethnicity into a single construct with those of specified Hispanic or Spanish origin as a separate population. While we recognize that this fails to capture important heterogeneity, this was not the primary population of interest for this study. It is expected that the greater Hmong population has little cross‐over with Spanish or Hispanic origin. Further, we chose the Non‐Hispanic White population as the reference population given its historical advantage and privilege within the United States and role as default reference population for NCDB epidemiological studies. For statistical purposes and ease of comparison, these categories were condensed into a modified race/ethnicity variable including subcategories of Hmong, Southeast Asian (encompassing variable options of Filipino, Vietnamese, Laotian, Kampuchean, and Thai), Non‐Hispanic White (NHW), Other Asian/Pacific Islander (encompassing all other recorded Asian and Pacific Islander sub‐populations), Non‐Hispanic Black, American Indian/Aleutian/ Eskimo, Spanish/Hispanic (a conglomerate variable derived from the NCDB ethnicity categories), and other/unknown. Other variables of interest included sex, site of cancer, analytic stage, population density of residence, average income, high school degree completion rate, primary insurance payor, Charlson–Deyo comorbidity score, treatment facility type, surgery completed at primary cancer site, chemotherapy completed, and immunotherapy completed. Radiation status was excluded given lack of a streamlined variable. For unknown data, a unique “unknown” category was included as a categorical option for each observed subject; no data was imputed.

### 2.2 Statistical analysis

Descriptive statistics of demographic, clinical, socioeconomic, and treatment variables were generated for all colon and rectal cancer cases, stratified by race/ethnicity. Chi‐squared tests were used to compare categorical variables, and one‐way ANOVA tests were used for comparison of means of continuous variables. The NCDB “analytic stage” variable was used for all analyses. Direct statistical comparison of age at time of diagnosis was completed using Wilcoxon rank‐sum (Mann–Whitney). Survival estimates were obtained using the Kaplan–Meier method; the log‐rank test was used to evaluate group comparisons. Two Cox‐proportional hazard models were generated to estimate hazard ratios (HRs) for OS. Within both models the primary independent variable of interest was race/ethnicity. The first model included demographic, socioeconomic, and clinical predictors as detailed above. Within the second model, treatment variables (surgical, chemotherapy, and immunotherapy completion) were included in addition to the variables used in the first model. Variables were assessed for covariance by direct variable correlation assessment; a correlation was noted between educational status and medical insurance type, but both variables were included given clinical context. The proportional hazard (PH) assumption was visually assessed via log–log (log minus log) plots given large number of cases.^23^ Variables grossly violating the PH assumption were adjusted for by stratification of the model allowing for differences in baseline hazards within the different strata.^24^ Of note, analytic stage appears to have potentially violated PH assumption, but models run with and without stratification based on analytic stage did not alter outcomes significantly. Analytic stage was included as a relevant variable in both models given known clinical significance with regards to survival. All models were further stratified based on disease site (colon, rectum). Sensitivity analyses was performed by using only cases with all variables recorded (complete‐case analysis with exclusion of cases with any unknown data). Statistical analysis was performed using STATA version 16.1,^25^ and a significance level of *p* < 0.05 was set for all statistical tests.

## 3 RESULTS

### 3.1 Patient population

A total of 881,243 cases of colon (*n* = 685,710; 77.8%) and rectal (*n* = 195,533; 22.2%) adenocarcinoma were identified in the NCDB between the years of 2004 and 2017. Of these, 120 (0.01%) individuals were identified as Hmong. Within the Hmong population, a total of 78 (65.0%) colon cancers and 42 (35.0%) rectal cancers were identified. Summary statistics of demographic, clinical, socioeconomic, and treatment variables for combined colon and rectal cancers are included in Table 1. Notably, 30.8% of Hmong individuals lived in the lowest income quartile zip‐codes, and over half (50.8%) lived in the zip‐codes with the highest percentage of individuals not completing high school degrees. A higher percentage of Hmong individuals had Medicaid health insurance (28.3%) compared to NHW individuals (3.7%) and a lower percentage (31.7%) had Medicare compared to NHW individuals (58.2%).

**TABLE 1:** Demographic, clinical, socioeconomic, and treatment characteristics of patients diagnosed with colon and rectal adenocarcinoma stratified by race/ethnicity.

	Hmong	Southeast Asian	Non‐Hispanic White	Other Asian/Pacific Islander	Non‐Hispanic Black	American Indian, Aleutian, or Eskimo	Spanish/Hispanic	Other/Unknown
Total cases (*n*)	120	6482	691,235	20,221	105,390	2814	43,640	11,341
Age at diagnosis, mean (SD)*	58.9 (17.4)	63.5 (13.3)	68.7 (13.5)	64.9 (13.0)	64.2 (13.2)	62.1 (13.0)	62.7 (14.2)	65.1 (13.9)
Sex (%)*								
Male	46.7	52.5	51.6	51.7	48.6	52.5	55.4	54.4
Female	53.3	47.5	48.4	48.3	51.4	47.5	44.6	45.6
Cancer location (%)*								
Colon	65.0	72.3	77.5	74.8	82.7	69.4	74.2	75.9
Rectum	35.0	27.7	22.5	25.2	17.3	30.6	25.8	24.1
Analytic stage (%)*								
0	<9.2	3.5	4.4	4.5	5.1	3.6	4.2	6.0
I	10.8	17.7	20.9	19.6	16.8	16.7	17.1	19.9
II	20.0	20.8	25.0	22.9	21.7	26.0	23.5	22.2
III	30.0	30.3	24.5	28.7	24.9	25.0	27.5	22.5
IV	>15.0	20.1	18.0	17.3	24.0	21.3	20.6	17.8
Unknown	15.0	7.6	7.2	7.0	7.5	7.4	7.0	11.6
Facility location (%)*								
Northeast	^a^	18.9	40.9	33.0	49.6	17.4	33.6	46.1
Midwest	50.0	18.9	28.4	10.9	20.3	21.0	8.2	23.7
South	^a^	6.0	14.7	7.0	22.0	19.7	22.5	11.1
West/Mountain	^a^	2.9	4.4	2.9	0.9	21.2	7.2	3.9
Pacific/Other	33.3	59.6	9.5	42.3	3.9	16.3	22.8	11.5
Unknown	14.2	3.9	2.1	4.0	3.2	4.4	5.7	3.7
Urban, rural classification (%)*								
Metro	97.5	95.8	79.9	95.2	88.7	51.0	93.3	87.2
Urban	^a^	2.0	15.2	2.3	8.3	34.9	4.8	8.4
Rural	^a^	0.2	2.1	0.1	1.2	11.1	0.4	1.0
Unknown	^a^	2.1	2.8	2.3	1.8	3.0	1.5	3.4
Median income by zip code (%)*								
<$40,227	>27.5	8.5	14.6	8.1	43.7	39.3	25.9	14.3
$40,227–$50,353	35.0	13.1	21.6	12.3	18.7	20.8	22.1	17.4
$50,353–$63,332	19.2	22.9	22.8	18.9	13.5	16.1	21.6	21.4
≥$63,333	9.2	45.3	33.2	53.3	15.6	14.2	22.5	39.0
Unknown	<9.2	10.2	7.9	7.5	8.4	9.6	7.9	7.8
No high school degree completion by zip code (%)*								
≥17.6%	50.8	32.7	15.4	22.8	39.4	31.6	50.8	20.7
10.9%–17.5%	15.8	20.4	24.7	17.5	29.3	27.3	19.2	22.4
6.3%–10.8%	19.2	23.0	28.2	24.9	15.8	20.5	13.9	24.7
<6.3%	^a^	13.8	24.0	27.3	7.2	11.4	8.3	24.5
Unknown	^a^	10.2	7.7	7.5	8.3	9.3	7.8	7.7
Insurance payor (%)*								
Not Insured	^a^	4.9	2.4	5.2	6.4	2.9	10.4	4.1
Private	35.0	42.4	33.3	41.4	33.1	27.0	34.5	40.9
Medicaid	28.3	13.0	3.7	11.1	10.6	11.7	13.9	7.4
Medicare	31.7	37.5	58.2	39.6	46.8	41.6	38.2	42.7
Other government	^a^	1.0	0.9	0.8	1.1	14.3	0.6	1.2
Unknown	^a^	1.2	1.5	2.0	2.1	2.5	2.4	3.7
Charlson–Deyo Score (%)*								
Score of 0	71.7	75.9	69.7	77.4	68.7	66.9	72.5	76.2
Score of 1	19.2	18.1	20.6	16.7	21.5	21.9	19.8	17.3
Score of 2	^a^	3.7	6.4	3.8	6.1	7.5	4.8	4.4
Score of 3	^a^	2.3	3.3	2.0	3.7	3.8	2.9	2.1
Facility type (%)*								
Community cancer program	<10.0	13.6	12.5	11.8	9.0	17.2	9.1	7.1
Comprehensive community cancer program	45.8	40.8	47.4	35.6	35.8	52.0	37.1	33.8
Academic/Research program	25.0	30.9	24.8	38.3	38.1	20.4	34.5	38.1
Integrated network ancer program	9.2	10.9	13.2	10.3	13.9	6.0	13.6	17.4
Unknown	>10.0	3.9	2.1	4.0	3.2	4.4	5.7	3.7
Surgical treatment (%)*								
No surgery at primary site	31.7	17.7	15.2	15.7	20.3	18.0	18.6	20.1
Surgery at primary site	68.3	82.2	84.6	84.0	79.5	81.4	81.1	79.4
Unknown	0.0	0.2	1, 0.2	0.3	0.2	0.6	0.3	0.6
Chemotherapy treatment (%)*								
No chemotherapy	>53.3	47.1	57.4	51.4	53.4	49.4	47.7	56.7
Chemotherapy	37.5	49.8	39.4	44.2	42.4	47.4	47.7	36.9
Unknown	<9.2	3.1	3.2	4.4	4.2	3.2	4.7	6.4
Immunotherapy treatment (%)*								
No immunotherapy	99.2	96.6	96.8	95.8	95.8	95.8	96.2	95.5
Immunotherapy	^a^	2.8	2.2	2.7	2.9	3.6	2.8	2.1
Unknown	^a^	0.6	1.0	1.5	1.3	0.6	1.0	2.4

^a^ Cell size <10 cases, data suppressed.

* Statistically significant with *p*‐value <0.05.

### 3.2 Age at diagnosis and analytical stage

**FIGURE 1:**
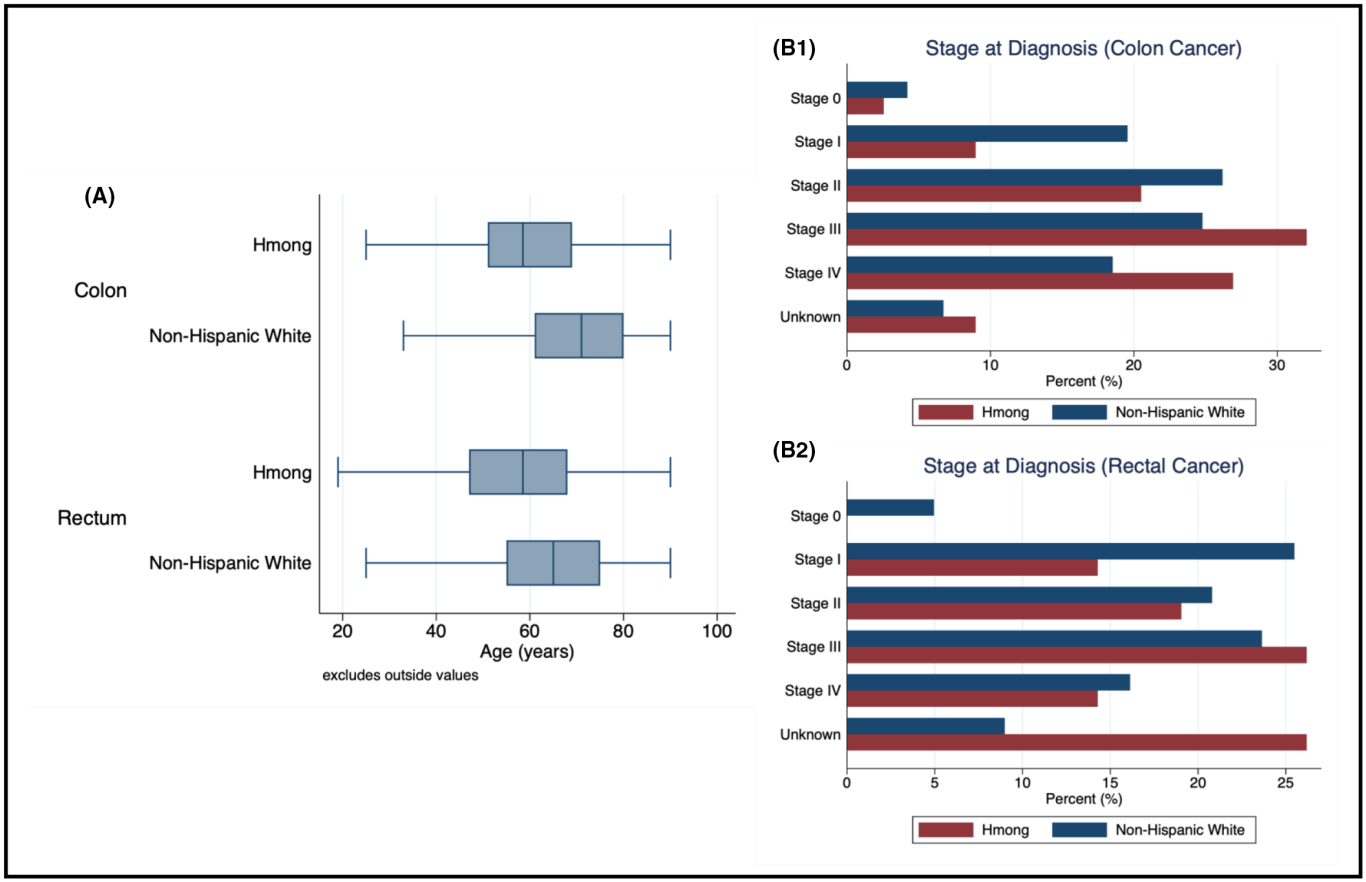
(A) Box plot demonstrating the age of distribution at the time of diagnosis for Non‐Hispanic White and Hmong populations stratified by cancer location. Wilcoxon rank‐sum (Mann–Whitney) test: *p* = 0.002 and Rectum: *p* = 0.002, (B1) and (B2). Distribution of analytic stage at the time of diagnosis for patients diagnosed with colon and rectal cancer, respectively. Exact percentages suppressed due to low‐case count.

When stratified by cancer location, the average age of diagnosis for colon cancer in NHW individuals was 68.6 years (median 71, SD 13.5 years) compared to 58.9 years (median 58.5, SD 17.0 years) for Hmong individuals (*p* < 0.001). The average age of diagnosis for rectal cancer in NHW individuals was 64.3 years (median 65, SD 13.6 years) and 58.8 years (median 58.5, SD 18.3 years) for Hmong individuals (*p* = 0.002). Box plots demonstrating distribution of age at diagnosis are included in Figure 1A.

The distribution of analytic stage differed between the Hmong population and the reference NHW population (*p* = 0.001; Figure 1B), and when excluding cases with an unknown stage, Hmong patients had a higher distribution of analytic stage (trending towards stage III and IV) compared to NHW patients (*p* = 0.02). In aggregate, 61.8% of Hmong individuals compared to 45.8% of NHW individuals with known analytic were diagnosed with stage III or IV CRC compared to 0, I, or II (*p* = 0.001). Of Hmong patients diagnosed with colon cancer, most were reported as stage III (32.05%) compared to most NHW patients being diagnosed with stage II colon cancer (26.19%). Within the rectal cancer populations, most NHW patients were diagnosed with stage I rectal cancer (25.49%). Over a quarter of Hmong individuals were diagnosed with stage III rectal cancer (26.19%) with the same proportion of Hmong individuals diagnosed at an unknown stage (26.19%). The proportion of Hmong patients with an unknown stage is significantly higher compared to the proportion of NHW population with unknown stage (8.99%, *p* < 0.001). Further comparisons are limited by low case count and high proportion of unknown stage for Hmong patients diagnosed with rectal cancer.

### 3.3 Survival analysis

**FIGURE 2:**
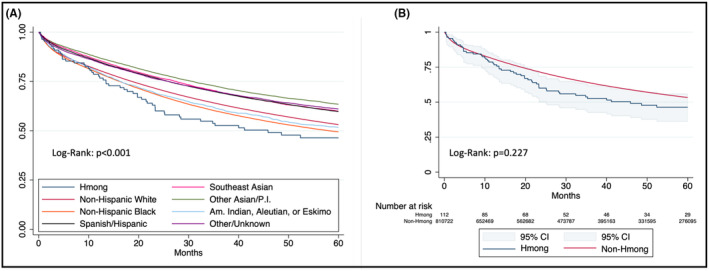
Kaplan–Meier curve of 5‐year OS estimates of patients diagnosed with colorectal adenocarcinoma across (A) all race/ethnicity groups and (B) binary Hmong ethnicity status with added confidence interval bands and risk table.

There appears to be a difference in unadjusted OS between all race/ethnicity groups at 60 months of follow‐up (*p* < 0.001; Figure 2A). However, when comparing Hmong ethnicity against an aggregated group encompassing all other race/ethnicities, no statistically significant difference in OS is evident despite separation of KM curves (*p* = 0.227; Figure 2B). When accounting for potential confounders, there was no association between OS and Hmong ethnicity, compared to the reference NHW population (HR 1.22 [0.94–1.58]; *p* = 0.13; Table 2). Additionally, no survival association was evident when further controlling for treatment status between the Hmong and reference NHW population (HR 1.00 [0.77–1.33]; *p* = 0.998; Table 2). Sensitivity analysis (complete‐case analysis) utilizing cases without any unknown data did not result in different conclusions.

**TABLE 2:** Multivariable Cox‐proportional hazard models.

Race/Ethnicity	Model 1: Adjusted for demographic, clinical, and socioeconomic factors				Model 2: Adjusted for demographic, clinical, socioeconomic, and treatment factors			
	Model stratified on cancer location, facility location, and facility type				Model stratified on cancer location, facility location, facility type, chemotherapy completion, and immunotherapy completion			
	Hazard ratio	[95% confidence interval]		*p*‐value	Hazard ratio	[95% confidence interval]		*p*‐value
Non‐Hispanic White	[Reference]				[Reference]			
Hmong	1.22	0.94	1.58	0.13	1.00	0.77	1.33	0.998
Southeast Asian	0.86	0.82	0.89	0.001	0.84	0.81	0.88	0.001
Other Asian/Pacific Islander	0.77	0.75	0.79	0.001	0.77	0.75	0.79	0.001
Non‐Hispanic Black	1.10	1.09	1.11	0.001	1.05	1.04	1.07	0.001
American Indian, Aleutian, or Eskimo	1.12	1.06	1.18	0.001	1.11	1.05	1.17	0.001
Spanish/Hispanic	0.82	0.81	0.84	0.001	0.80	0.79	0.82	0.001
Other/Unknown	0.84	0.81	0.86	0.001	0.80	0.77	0.82	0.001

## 4 DISCUSSION

In this study, we sought to summarize several traits as well as describe the survival of the Hmong population diagnosed with CRC using a national sample. The most striking finding in our study is a younger average age by nearly a decade at the time of diagnosis of CRC in the Hmong population as compared to the larger NHW population across a national sample. This reflects our community partners' lived experiences. We observed no association with overall‐survival in the Hmong population compared to the NHW population when controlling for a variety of demographic, socioeconomic, clinical, and treatment factors. However, a trend towards lower OS in the Hmong population that did not achieve statistical significance given the small sample size is possible.

The underlying cause of younger age at the time of CRC diagnosis in the Hmong population is unclear and could be related to health‐seeking behaviors, socioeconomic factors, population dynamics, innate NCDB limitations, and/or other unknown influences. Within this analysis, over 27% of the Hmong population was in the lowest annual income quartile, and prior work suggests worse outcomes of CRC are observed in lower socioeconomic groups.^26^ This may be in part due to an association between lower socioeconomic status and lifestyle risk factors of early onset CRC such as obesity, diabetes, food insecurity, processed and red meat consumption, and alcohol/tobacco dependance.^26^, ^27^, ^28^, ^29^, ^30^ Though many of these lifestyle risk factors have yet to be directly described in the Hmong population and remains an open area for investigation. Hmong individuals may utilize non‐COC facilities and communal resources for care; the enrichment of younger cases observed may be a self‐selection bias of those wishing to receive specialized care. However, preliminary data (unpublished) from the state of WI cancer registry—which incorporates non‐COC accredited facilities—demonstrates a similar trend towards younger ages at the time of diagnosis in the state of Wisconsin without differences in OS. The authors also acknowledge that the NCDB is comprised of patient data from CoC‐accredited facilities; therefore a baseline level of quality care is presumed. It is possible that differences in OS exist for the Hmong population presenting to non‐CoC facilities, yet further research is needed to assess this hypothesis. With regards to national age distribution in 2017, the US Hmong population is mostly comprised of younger individuals with the median Hmong age around 25.1 years compared to 38.1 years for the greater US population.^3^, ^31^ The earlier age of onset may be directly related to a younger immigrant population.

National trends suggest an increasing incidence of early‐onset CRC (before the age of 50) leading to recommendations for earlier screening age which may be particularly important for the Hmong population given our findings of a younger average age at the time of diagnosis. Interestingly, the reference NHW population had the highest age at diagnosis. Yet, the Southeast Asian and Other Asian/Pacific Islander groups had statistically improved OS compared to NHW population within our models (and observed as separation of the Kaplan Meier curves). This is an interesting finding that warrants further investigation.

This analysis also suggests a trend towards higher stage at diagnosis within the Hmong population. Prior work assessing health‐seeking behaviors in Hmong Americans noted decreased utilization of cancer screening tests due to decreased health insurance access, language barriers, and lower health literacy.^32^ Recent work by Jain et al. described colon cancer trends with a focus on time to surgery and analytic stage in a disaggregated Asian population (with Hmong as a unique subpopulation) using NCDB data.^33^ Their work did not identify a statistical difference in time to surgery (in stage 0–III colon cancer cases) compared to a NHW population but noted a trend towards a higher stage in the Hmong population, like our findings. The high proportion of unknown stage of disease in the Hmong population, particularly within rectal cancer, was unexpected. Within the Surveillance, Epidemiology and End Results (SEER) national database, an unknown stage at the time of diagnosis was more common in more lethal malignancies such as liver, esophageal, and pancreatic cancer.^34^ Further, in CRC cases identified within the SEER database, patients with an unknown stage at diagnosis were more likely to be older, African American, and female.^35^ These observations along with other smaller population‐based studies suggest that an unknown stage of malignancy in population‐based studies is more common in vulnerable populations.^34^, ^35^, ^36^, ^37^ Thus, the high proportion of unknown stage in the Hmong population may be associated with overall worse cancer outcomes, but there is insufficient information to explain the underlying reason for this finding.

This study has several limitations, many of which are innate to NCDB‐based studies, including: missing data, limited number of variables (i.e., possible unmeasured confounding bias), inability to calculate age‐adjusted statistics (i.e., incomplete coverage of all captured cancer cases nationally), and inconsistencies in ethnicity data capture.^38^, ^39^, ^40^ We have attempted to mitigate potential confounding through advanced statistical methods. We were unable to account for several lifestyle metrics such as diet, alcohol and tobacco consumption, which may be of particular importance for the diagnosis of colon and rectal cancers, as these were not included within these data. The small Hmong population size raises concern for overfitting of our adjusted model and may not capture statistical differences in overall‐survival. Race and ethnicity are based on direct identification, reported by hospital systems and ideally collected from patient self‐reported answers. However, it is possible that the Hmong population has been underestimated. Foote et al. noted that only a minority of hospitals (26%) in the Wisconsin State Cancer Registry reported “Hmong” as a unique population.^40^ Regardless, Hmong patients presenting to COC‐accredited facilities are on average much younger than their NHW counterparts which provides opportunities for both intervention and further study.

## 5 CONCLUSIONS

Hmong patients presenting to COC‐accredited facilities are diagnosed with CRC at a younger age and seemingly higher stage than NHW patients. Despite this, there does not appear to be an association with decreased OS among Hmong individuals diagnosed and treated for CRCs. Further work should focus on disseminating these findings with community members and working towards outreach and community‐healthcare‐academic partnerships founded on the priorities of those affected, the Hmong community. Particular focus should be given to understanding and reducing barriers to screening, prevention and cancer‐related care.

## AUTHOR CONTRIBUTIONS

**Margaret R. Walker:** Conceptualization (equal); data curation (equal); formal analysis (lead); investigation (equal); methodology (equal); validation (equal); visualization (lead); writing – original draft (lead); writing – review and editing (lead). **Kha Lor:** Writing – original draft (equal); writing – review and editing (equal). **Roberto J. Vidri:** Methodology (equal); supervision (equal); validation (equal); writing – review and editing (equal). **Kajua B. Lor:** Conceptualization (equal); writing – review and editing (equal). **John M. Hampton:** Methodology (equal); validation (equal); writing – review and editing (equal). **Cinthya Maldonado:** Writing – review and editing (equal). **Andrea M. Schiefelbein:** Writing – review and editing (equal). **Noelle K. LoConte:** Conceptualization (equal); funding acquisition (equal); resources (equal); supervision (lead); writing – review and editing (equal).

## CONFLICT OF INTEREST STATEMENT

None of the authors have direct conflicts of interest for this study. Noelle K. LoConte has participated in advisory boards for Personal Genome Diagnostics and Abbvie in the past 3 years.

## PRECIS

Hmong patients presenting to Commission on Cancer‐accredited facilities are diagnosed with colon and rectal cancers at a younger age than Non‐Hispanic White patients. However, when accounting for potential confounders, there was no association between overall survival and Hmong ethnicity, compared to the reference NHW population.

## Data Availability

The National Cancer Database (NCDB) is a joint project of the Commission on Cancer (CoC) of the American College of Surgeons and the American Cancer Society. The CoC's NCDB and the hospitals participating in the CoC's NCDB are the source of the de‐identified data used herein; they have not verified and are not responsible for the statistical validity of the data analysis or the conclusions derived by the authors. Restrictions apply to the availability of these data, which were used under license for this study. Data are available https://www.facs.org/quality‐programs/cancer‐programs/national‐cancer‐database/ with the permission of American College of Surgeons.
